# Influence of pre-pregnancy body mass index (p-BMI) and gestational weight gain (GWG) on DNA methylation and protein expression of obesogenic genes in umbilical vein

**DOI:** 10.1371/journal.pone.0226010

**Published:** 2019-12-03

**Authors:** Erika Chavira-Suárez, Angélica Jazmín Ramírez-Mendieta, Sofía Martínez-Gutiérrez, Paola Zárate-Segura, Jorge Beltrán-Montoya, Nidia Carolina Espinosa-Maldonado, Juan Carlos de la Cerda-Ángeles, Felipe Vadillo-Ortega

**Affiliations:** 1 Departamento de Bioquímica, Facultad de Medicina, Universidad Nacional Autónoma de México, Mexico City, Mexico; 2 Unidad de Vinculación Científica de la Facultad de Medicina, Universidad Nacional Autónoma de México en el Instituto Nacional de Medicina Genómica, Mexico City, Mexico; 3 Laboratorio de Medicina Traslacional, Escuela Superior de Medicina, Instituto Politécnico Nacional, Mexico City, Mexico; 4 Instituto Nacional de Perinatología Isidro Espinosa de los Reyes, Mexico City, Mexico; 5 Hospital General Dr. Enrique Cabrera, Secretaría de Salud CDMX, Mexico City, Mexico; University of Insubria, ITALY

## Abstract

Understanding the regulatory mechanisms that affect obesogenic genes expression in newborns is essential for early prevention efforts, but they remain unclear. Our study aimed to explore whether the maternal p-BMI and GWG were associated with regulatory single-locus DNA methylation in selected obesogenic genes. For this purpose, DNA methylation was assayed by Methylation-Sensitive High Resolution Melting (MS-HRM) technique and Sanger allele-bisulfite sequencing in fifty samples of umbilical vein to evaluate glucosamine-6-phosphate deaminase 2 (*GNPDA2*), peroxisome proliferator-activated receptor gamma coactivator 1 alpha (*PGC1α*), and leptin receptor (*LEPR*) genes. Correlations between DNA methylation levels and indicators of maternal nutritional status were carried out. Western blotting was used to evaluate protein expression in extracts of the same samples. Results indicated that *GNPDA2* and *PGC1α* genes have the same level of DNA methylation in all samples; however, a differential DNA methylation of *LEPR* gene promoter was found, correlating it with GWG and this correlation is unaffected by maternal age or unhealthy habits. Furthermore, leptin receptor (Lep-Rb) was upregulated in samples that showed the lowest levels of DNA methylation. This study highlights the association between poor GWG and adjustments on obesogenic genes expression in newborn tissues with potential consequences for development of obesity in the future.

## Introduction

Etiology of obesity lies on complex interactions between genetic, epigenetic and environmental factors [1–5]. Epidemiological and experimental evidence support that children and adult obesity is associated with parental health status and lifestyles that can modulate offspring developmental programming during the periconceptional period, fetal life, and early childhood [6–10]. Developmental programming of obesity may result from altered genetic expression as a fetal adaptive response to adverse intrauterine influences such as maternal diet or nutritional status during pregnancy [8,11–14].

Genome-wide association studies (GWAS) have identified several single nucleotide polymorphisms (SNPs) linking genetic traits with an increased risk for obesity development [15–17]. However, the study of environmental factors’ effects on gene expression to promote an obesogenic phenotype is still scarce. Epigenetic mechanisms such as DNA methylation have been proposed as mediators of adverse phenotypes [18–20]. Preliminary evidence suggests that maternal obesity can have significant effects on neonatal adiposity by altering offspring epigenome-wide DNA methylation [20]. Although many articles report DNA methylation variability in offspring tissues linked to obesogenic environments in pregnancy [21,22], it is still uncertain how DNA methylation variability is influenced by interindividual biological variation [23], ethnic and sex composition, and specific organ performance [24,25].

GWAS studies have identified common genetic variants associated with BMI of children and adults among European, Chinese, Japanese and Mexican populations [16,26–28], suggesting that there is a genetic risk for obesity [29]. Specific variants of *GNPDA2*, *PGC1α* and *LEPR* genes have shown a strong association with Mexican population’s BMI, increasing the risk of obesity [28,30–32], although the control mechanisms of obesogenic genes expression during early development are still unknown.

Nutritional status of pregnant woman is a major environmental contributor for fetal development and many studies have used p-BMI and/or GWG during pregnancy as indicators of the adequacy of the nutritional status [33,34]. We previously reported the use of these indirect indicators of maternal health in the assessment of differential methylation in newborns of a diet-responsive gene [35]. The current study aimed to explore whether maternal p-BMI and GWG are associated with a single-locus promoter methylation of *GNPDA2*, *PGC1α* and *LEPR* genes and their consequent protein expression in newborns.

## Materials and methods

### Subjects

Women recruited in their first trimester of pregnancy and attended for a prenatal care cohort at Hospital Materno Infantil Inguaran in Mexico City, participated in this secondary study (between January 2013 to July 2014), signing a letter of informed consent. Pregnant women who met the following criteria were eligible for the present study: age between 18 and 35 years, singleton and normal pregnancy clinically diagnosed, and delivering at 37–40 weeks of gestation. Pregnant women who met more than two vaginal infections, gestational diabetes or diabetes *mellitus*, preeclampsia, and preterm delivery, were excluded.

Data of birth weight (BW) and birth length (BL) were registered, and the umbilical cord was collected after placenta delivery. Medical and nutritional status, including anthropometric information (p-BMI and GWG) of patients during pregnancy were searched on the clinical records, such as the habits of smoking, alcohol, and drug consumption.

All procedures and data protection were approved by the Internal Review Board from the Mexico City Ministry of Health (Register 101-001-008-12).

### Sample size

The sample size was estimated using the BMI means and SD of two hundred and seven Hispanic reproductive-age women; the BMI values were approximately normally distributed [36]. The confidence level selected was 95% with an estimated proportion of 0.79 and a relative standard error of 6.5, calculating a result of 49 samples (https://www.abs.gov.au/websitedbs/D3310114.nsf/home/Sample+Size+Calculator).

### DNA isolation from umbilical cord vein

Total DNA from 50 mg of umbilical vein was extracted as follows: enzymatic tissue digestion was carried out with 500 μl/sample of solution [50 mM Tris-HCl, 100 mM EDTA, 100 mM NaCl, 1% SDS pH 8.0 and, 0.5 mg/ml of proteinase K (Invitrogen)] and it was incubated at 50°C overnight. The following day, the digested sample was centrifugated at 2,400 x g during 5 min to rescue the pellet in a new DNasa/RNasa-free microfuge tube and homogenized in 500 μl of TRIzol reagent (Invitrogen), and incubated at room temperature for 5 min. Ten μl of chloroform was added, mixed, incubated at room temperature for 3 min and, centrifuged at 11,200 x rpm for 15 min at 4°C. DNA precipitation was followed by addition of 100% ethanol and centrifugation at 4,700 x g for 5 min at 4°C. DNA pellet was rinsed three times, adding 500 μl of sodium citrate 0.1M/10% ethanol, incubated at room temperature 30 min and, centrifuged at 2,000 x g for 5 min at 4°C. Last rinsing was made with 400 μl of 75% ethanol, incubated 15 min and centrifuged. DNA was dissolved in 25 μl of TE buffer and incubated at 45°C for 1 h. DNA quality was assessed by measuring the absorbance at 260/280 nm (1.7–2.0) and was quantified with a Thermo Scientific Nanodrop 2000c Spectrophotometer (Thermo Scientific). DNA integrity was verified by 1% agarose gel electrophoresis.

### Primer designs and MS-HRM assay

Promoter sequences of *PGC1α*, *GNPDA2*, and *LEPR* genes were obtained from the UCSC Genome Browser database (version of Feb 2009 GRCh37/hg19 assembly). The selection of study sequences for each gene was based on chromosomes’ CpG island regions located near the transcriptional start site (TSS) and containing DNA binding site from conserved transcriptional factors (TF). The primers were designed according to the MS-HRM technique conditions [37] (Table 1).

**Table 1:** Characteristics of the target gene sequences and primers condition.

Characteristics	*PGC1α*	*GNPDA2*	*LEPR*
**Forward**	5’TAGGTTCGTTTTGATTTGGGTAGT3’	5’ATTCGGATGTAGATAAAGGCGTAG3’	5’TTCGCGAGTTAGGGGAGGAG3’
**Reverse**	5’TCTCGCCCTCTCGCTTCC3’	5’CTCCTCGCGTCTCACCTCAA3’	5’ACCCGACCTCGCTACTCAAA3’
**Primer annealing temperature**	59°C	54°C	54°C
**Chromosome** **(# of bases)**	Chr4:24,474,340–24,474,473 (134)	Chr4:44,728,592–44,728,691 (100)	Chr1: 65,886,000–65,886,146 (147)
**Conserved TF Binding Site**	EGR1-3, AHRARNT2	-	-
**TF Chip-seq (ENCODE March 2012)**	ZNF263, RBBP5, RUNX3, POLR2A, MYC, TCF7L2, RELA, E2F1, SIN3A, PAX5, BACH1, USF1, MAX.	POLR2A, REST, RBBP5, PHF8, HMGN3, YY1, KDM5B, MYC, MAX, E2F6, PML, GATA1, CTCF, TAF1, CBX3, SETDB1, GABPA, HDAC2, GATA2, TEAD4, RELA, UBTF, SIN3AK20, E2F4, NFYB, MAZ, CHD2, NR2C2, FOXP2, E2F1, TCF7L2, CREB1, POU2F2, RCOR1, USF2, USF1, ELF1, ATF3, IRF1, GATA3, RAD21, TBP, JUN, PAXS, GTF2B, NRF1, FOS, RUNX3, SPI1	POLR2A, MYBL2, MAZ, TAF1, TBP, MXI1, MYC, CHD2, HMGN3, EGR1, TFAP2C, CCNT2, STAT1, TEAD4, SP1, IRF1, NRF1

Forward and reverse primer sequences for each gene were designed and previously annealing-temperature optimized for MS-HRM assay conditions. The specific locus of each gene falls in a conserved TF binding-site or in a TF Chip-seq pipeline. Locations in chromosomes were downloaded at the UCSC Genome Browser database (Feb 2009 GRCh37/hg19 assembly version). TF: transcriptional factors.

Bisulfite conversion of umbilical vein DNA samples and methylation standard controls (500 ng/sample) were performed using the EZ DNA Methylation-Gold Kit (Zymo Research), according to the manufacturer´s protocol. Bisulfite-treated methylated and unmethylated standard controls (5ng/μL) were mixed at 50%, 10%, and 1% proportions to create a range of allele dilutions that were used to develop a methylation standard curve as a template for interpolation of values from bisulfite-treated DNA samples.

MS-HRM assays were first optimized to adjust the primers’ annealing temperature using the bisulfite-treated standard curve dilutions (S1 File). Then, conditions for the site-specific analysis of the *PGC1α*, *GNPDA2*, *LEPR* gene promoters were set as follows: 1) for amplification, 10 min at 95°C followed by 45 cycles of 10 s at 95°C, 10 s at the primer annealing temperature shown in Table 1, and 15 s at 72°C, 2) for high-resolution melting analysis, 1 min at 95°C, 5 s at 72°C, and continuous increase to 95°C with 50 acquisitions/C and, 3) a cooling setting of 30 s at 40°C. All reactions were performed three times in duplicate in white 96-well plates using a Light Cycler 480 Instrument (Roche).

End products of MS-HRM assays were first analyzed by Gene Scanning software (Roche), normalizing the fluorescence intensity of raw melt curves and aligning the normalized curves with a temperature shift. Then, melting peaks obtained by Tm calling option were generated from the negative first derivative of normalized curves and those data were exported to GraphPad Prism v6.0 for further analysis after the graphs were plotted. Normalized and temperature-shifted difference curves were used to obtain the area under the curve (AUC), generating the 100% methylation standard curve to the horizontal axis for determining the degree of methylation of each DNA samples [38].

### Direct sequencing

PCR amplification was carried on before Sanger sequencing with the end products of MS-HRM assays and the same MS-HRM primers, obtaining after amplification mixed templates. Sanger sequencing were performed with the BigDye Terminator v1.1 Cycle Sequencing Kit and using an ABI prism 370 DNA sequencer (Applied Biosystems). Nucleotide sequences were aligned using Mega 7.0 and manually adjusted in the text editor. Initial identification of the sequences was made after performing BLAST searches of the NCBI database. The electropherogram quality was visualized with 4Peaks X 1.7.1, and CpG highlighting was simplified using the BiQ Analyzer software tool.

### Protein extraction and western blotting

For 1 day, fifty milligrams of umbilical vein were treated with lysis buffer [50 mM Tris HCl, 1% Nonidet P-40, protease inhibitor cocktail, pH 7.4 (Sigma Aldrich)] at 37°C. Next day, samples were homogenized and incubated on ice for 1 h, centrifuged at maximum speed at 4°C for 10 min, and the supernatant was obtained. Fifty micrograms of protein in Laemmli buffer (1:1) was loaded in 15% SDS-PAGE gels and transferred to a PVDF (Thermo Scientific Pierce). The membrane was blocked with 5% non-fat dry milk dissolved in TBST for 30 min at room temperature. Blots were incubated with rabbit anti-human LEPR [1μg/ml (Abcam, ab104403)] or rabbit anti-β-actin-HRP [1:5000 (Abcam)] overnight at 4°C, washed with TBST, and incubated with secondary goat anti-rabbit- HRP [1:2000 (Abcam)] for 2 h at room temperature. Image acquisition and densitometry analysis were performed using Image Lab software version 5.2.1 build 11 (Bio-Rad).

### Statistical analysis

Clinical information of the subjects involved in the study is reported as mean ± SD, and unhealthy habits is reported in percentages. Correlation analysis between BW and BL with p-BMI and GWG was performed with a simple linear regression. Methylation status was obtained using the mean of AUC ± SD from all reactions in normalized and temperature-shifted difference plots. The mean of AUC showing normal Gaussian distribution assessed using Shapiro–Wilk test were compared between methylation groups through an unpaired t-test with equal SD and Welch’s correction. The correlation analysis of the p-BMI, GWG, BW, and BL with the methylation status (AUC data) was completed using a v2 test and a Pearson correlation with linear regression; correlation between GWG and *LEPR*’s AUC was tested by a multiple regression model adjusted by least squares, using maternal age and unhealthy habits as confounder variables. Protein expression is reported as median with interquartile ranges (IQRs) and evaluated with a Mann–Whitney test (non-parametric unpaired t-test). Different values (p < 0.05) were corrected by Tukeys’ multiple comparisons test when it was required. The statistical and plots software used was GraphPad Prism v6.0 and JMP v11.0.

## Results

### Characteristics of studied subjects

Fifty pregnant women were recruited for this study and their clinical data were recorded (Table 2). Briefly, pregnant women showed a natural fertility age with low heights compared with those of other populations [39], and a marked overweight. Some participants occasionally smoked, consumed alcohol and/or used illicit drugs without affecting the birth BW and birth BL of newborns. BW was positively correlated with maternal p-BMI (r = 0.367, p = 0.009) but not with GWG. BL was not correlated with maternal p-BMI, nor for GWG (S1 Fig).

**Table 2:** Clinical characteristics of fifty pregnant women and their newborns from Mexico City.

Clinical feature	Mean ± SD	CI 95%
Maternal age (years)	29.48 ± 6.364	27.67–31.29
Pre-pregnancy weight (kg)	69.28 ± 16.31	64.64–73.92
Maternal height (cm)	156.3 ± 6.986	154.4–158.3
Weight at delivery (kg)	76.51 ± 14.64	72.35–80.67
p-BMI (kg/m^2^)	28.61 ± 6.415	26.78–30.43
Total GWG (kg)	7.036 ± 5.307	5.528–8.544
Gestational week at delivery	38.25 ± 0.8591	38.01–38.50
BW (g)	3140 ± 422.6	3020–3260
BL (cm)	48.95 ± 2.241	48.31–49.59
**Unhealthy habits**	**Positive % (N)**	**Negative % (N)**
Tobacco smoker (active or passive)	34 (17)	66 (33)
Alcohol intake	14 (7)	86 (43)
Use of illicit drugs	6 (3)	94 (47)

Descriptive results are expressed as the means ± SD, indicating the number of participants (N) and the corresponding percentage (%). Qualitative data are available by the presence/absence of consumption habits or complications during pregnancy. p-BMI: pre-pregnancy body mass index; GWG: gestational weight gain; BW: birth weight; BL: birth length; SD: Standard Deviation; CI: Confidence Interval.

### Site-specific gene promoter methylation status and its correlation with maternal nutritional status

**Fig 1:**
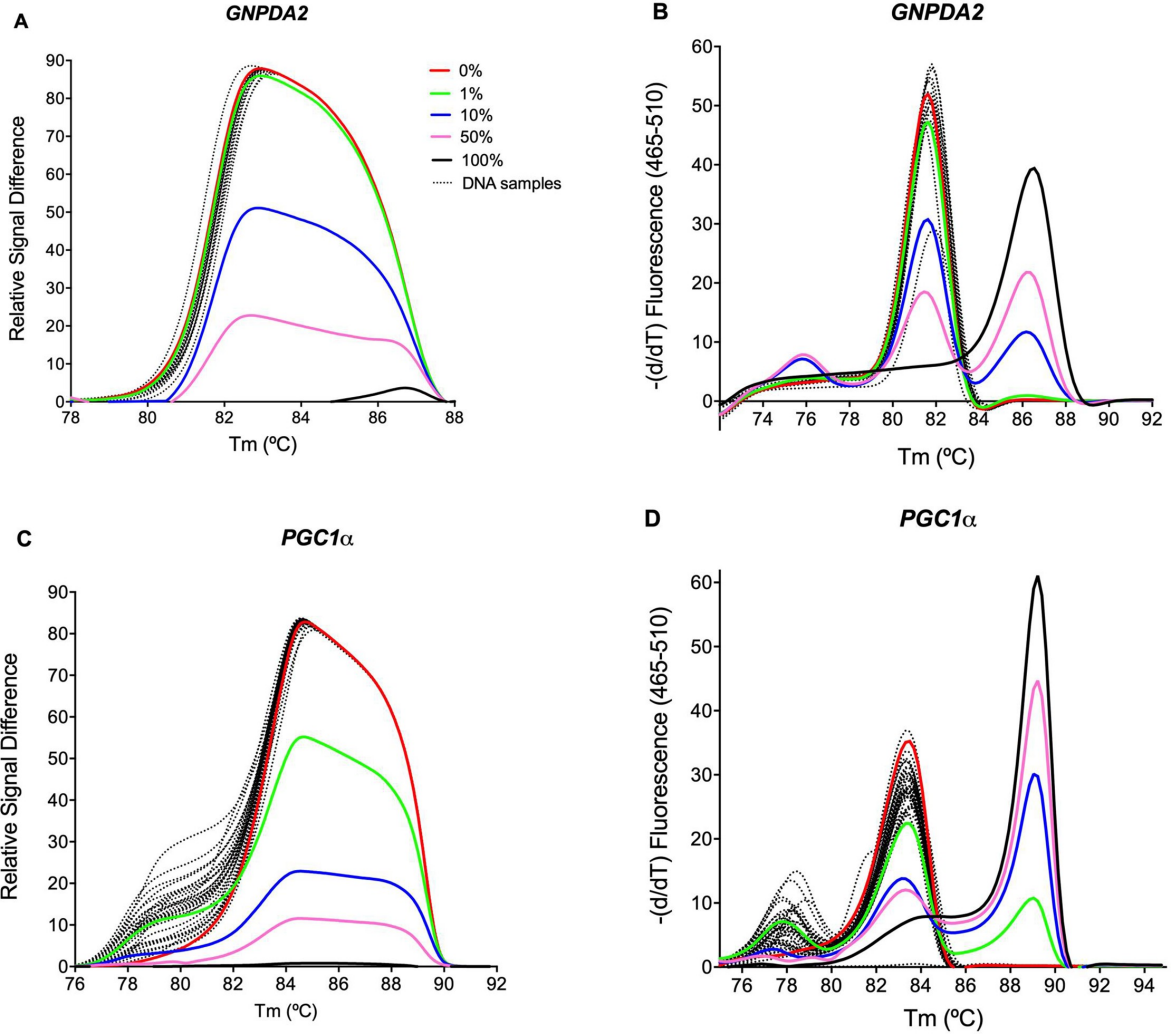
DNA methylation status in a single locus of *GNPDA2* and *PGC1α* promoters from newborns. (A) and (C) are representative difference plots of normalized and temperature-shifted gene promoters showing the reference curves of methylation as 0%, 1%, 10%, 50%, and 100% methylated universal standard dilutions. (B) and (D) are the gene promoter methylation statuses represented by melting peaks plots.

To evaluate methylation status of *GNPDA2*, *PGC1α* and *LEPR* promoters in newborns, fifty bisulfite-modified DNA samples from umbilical vein were analyzed. We obtained the melting profiles of a locus-specific assessment of the *GNPDA2*, *PGC1α* and *LEPR* and estimated the degree of methylation compared with a human control set of defined mixtures of methylated and unmethylated DNAs. Those profiles were represented by melting peaks and normalized temperature-shifted curve plots to obtain the AUC. For each sample, *GNPDA2* (Fig 1A and 1B) and *PGC1α* (Fig 1C and 1D) promoters did not show differences between human control set of 0% and bisulfite-modified DNA samples.

**Fig 2:**
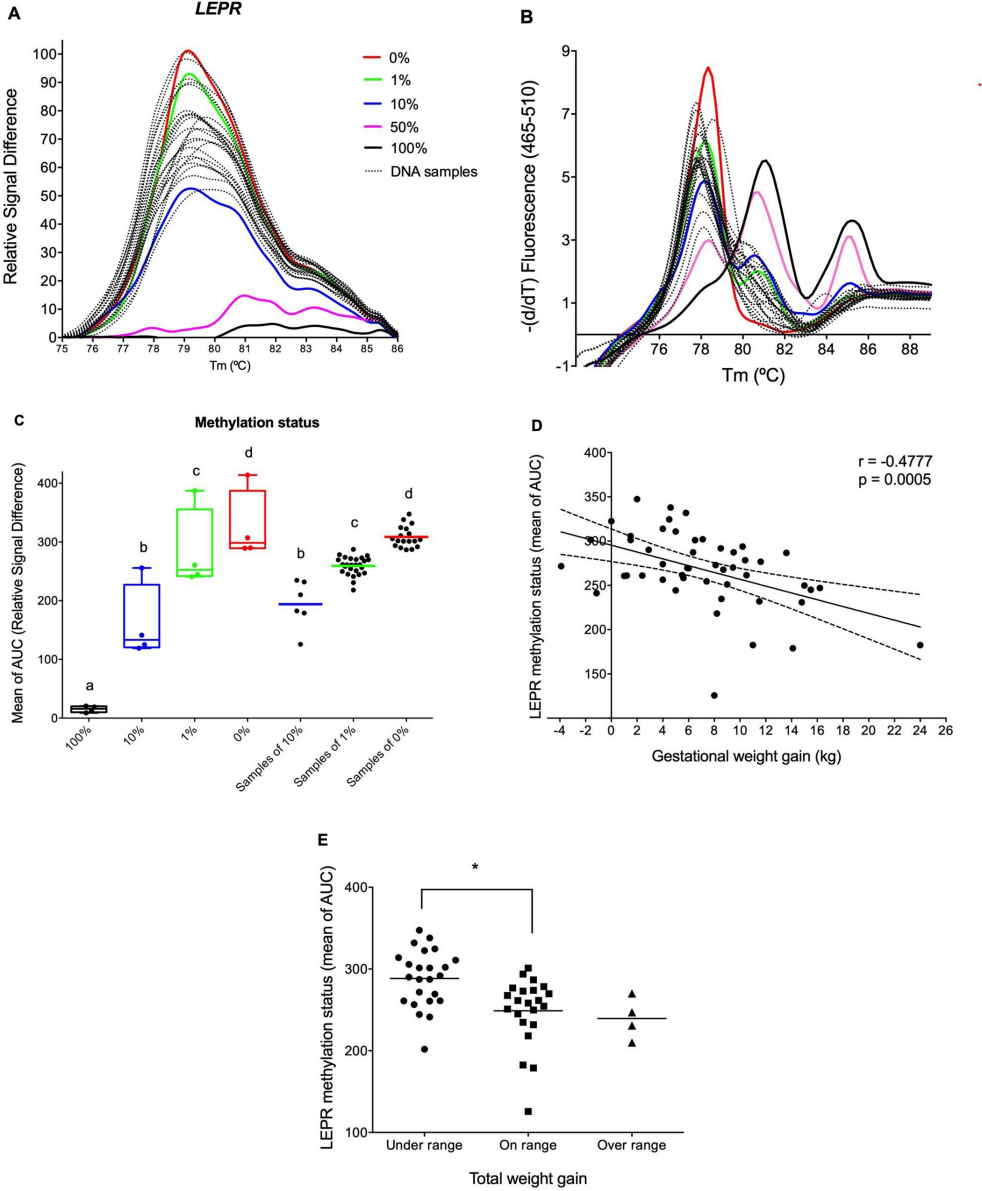
GWG impacts on DNA methylation variability in a single locus of the *LEPR* promoter. (A) Normalized and temperature-shifted difference plot. (B) Melting peaks plot of *LEPR* promoter methylation status, comparing it with the reference curve from human control sets as 0%, 1%, 10%, 50%, and 100% methylated universal standard dilutions. (C) *LEPR* methylation status expressed by the mean of AUC (DNA samples of 10% = 194.4 ± 40.9; of 1% = 259.3 ± 15.3; of 0% = 308.6 ± 17.8) and its comparison with the control sets (100% = 15.3 ± 5.3; 10% = 160.3 ± 64.3; 1% = 283.3 ± 69.7; 0% = 325.1 ± 59.9). Different letters over each box and whiskers show statistical difference data (p < 0.05) obtained by one-way ANOVA post-Tukey’s multiple comparisons test. (D) Negative correlation between *LEPR* methylation status and GWG obtained by Pearson’s correlation test. (E) Differential *LEPR* methylation status in the newborns that were classified by maternal total weight gain according to recommendations of the IOM. Statistical difference data (*p < 0.01) was obtained by Kruskal-Wallis test corrected by Dunn’s multiple comparisons test.

In this study, mean values of the AUC were used as indirect and inverse measure of the DNA methylation status (For example: AUC’s control set of 0% = 294.5 ± 133; AUC’s control set of 100% = 10.9 ± 13.3). To determine the methylation status of a specific locus of the *LEPR* promoter, we calculated the standard deviations of the difference between mean values of the AUC of 10%, 1% and 0% control sets under the independent assumption, showing that 52% of DNA samples corresponded to the control set of 1%, the 37% of DNA samples corresponded to the control set of 0%, and only the 12% corresponded to the control set of 10% (Fig 2A–2C).

Correlation analysis was realized using maternal nutrition status indicators and a single locus of *LEPR* promoter methylation status. *LEPR* methylation status in umbilical vein was negatively correlated with GWG (Fig 2D) without being affected by maternal age nor unhealthy habits (r^2^ = 0.29, adjusted r^2^ = 0.14, f-value = -2.98, p = 0.005). The results also indicate that the lowest state of *LEPR* promoter methylation is correlated with mothers who gained less weight, and vice versa (this is demonstrated by the highest mean of AUC). No correlations were found between *LEPR* methylation and p-BMI, BW or BL (r = 0.23 and p = 0.11, r = 0.13 and p = 0.34, r = 6 x 10^−4^ and p = 0.99, respectively).

For more studies, we classified the weight gain of mothers in three ranges (under, on, and over range), according to the guidelines revised by the Institute of Medicine (IOM) that are based on p-BMI ranges for women with underweight, normal weight, overweight, and obesity independently of their age, parity, smoking history, race, and ethnic background [39]. This kind of classification let us identify the umbilical vein samples from newborns exposed to under range GWG, obtaining the lowest *LEPR* methylation status compared to newborns exposed to on range GWG. No statistical differences were observed in *LEPR* methylation status between on range and over range GWGs, probably for the short number of over range mothers (Fig 2E).

In order to evaluate the influence of unhealthy habits during pregnancy on the *LEPR* methylation status of umbilical vein samples, we classified the women who smoked, consumed alcohol or used illicit drugs during pregnancy by three status of *LEPR* Methylation (0%, 1% and 10%) and were compared further with the non (smoker/alcohol/drugs) groups.

**Fig 3:**
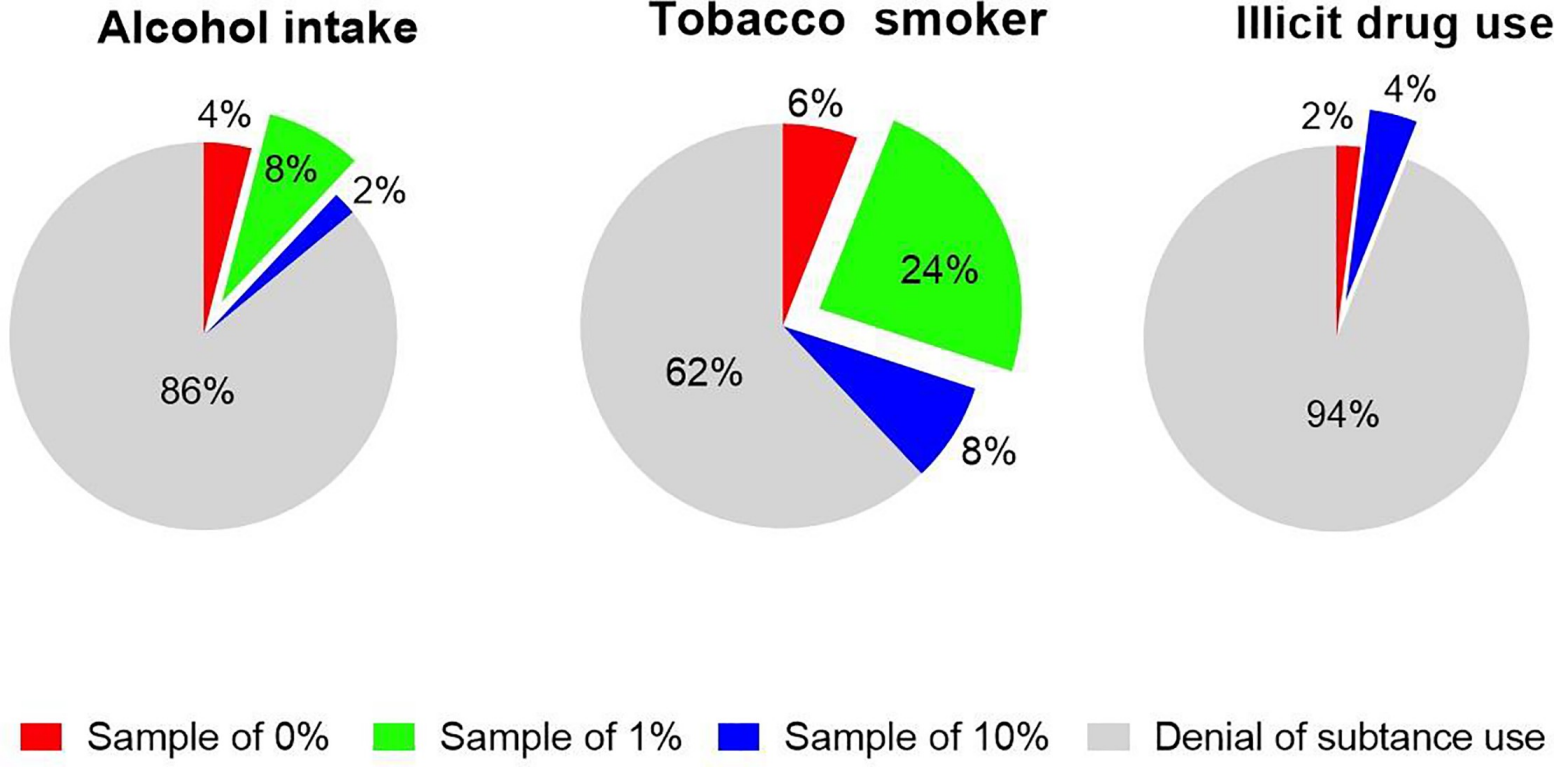
*LEPR* methylation status variability in umbilical vein of newborns exposed to healthy and unhealthy maternal consumption habits. Pie charts show the percentage of pregnant women who smoked, consumed alcohol, used drugs, and denied substance use, according to *LEPR* promoter methylation status.

Our findings showed that the frequencies of different *LEPR* methylation status between those groups healthy and unhealthy have not statistical differences between them (χ^2^ = 5.706, df = 6, p = 0.46). Nevertheless, most of the women who smoked (38%) and consumed alcohol (14%) had a methylation status of 1% (Fig 3).

### Validation of site-specific LEPR promoter methylation status

Eleven of MS-HRM products (around 20% of total DNA samples) were randomly selected to validate the results of umbilical vein DNA methylation status by Sanger sequencing. Sequenced samples were aligned with *LEPR* promoter wild-type sequence (WT) and with hypothetical *LEPR* methylated sequence (MS), both of them downloaded from NCBI database (Reference Sequence: NG_015831.2) or MethPrimer program [40], respectively. Human DNA methylation control sets of 0%, 1% and 10% were also sequenced to use them as methylation status references of direct sequencing [41] and to facilitate the methylated CpGs alignments of each sequenced sample (S2 File A), showing that non-methylated CpGs were modified into TpGs and methylated CpGs were conserved in MS-HRM, such as expected. Sequencing-alignments also revealed that other bases underwent transformation due to a prolonged sodium bisulfite exposure, showing a low-frequency modification in CpG sites into GpG or ApG, and some G bases were transformed into A or T base (S2 File B).

**Fig 4:**
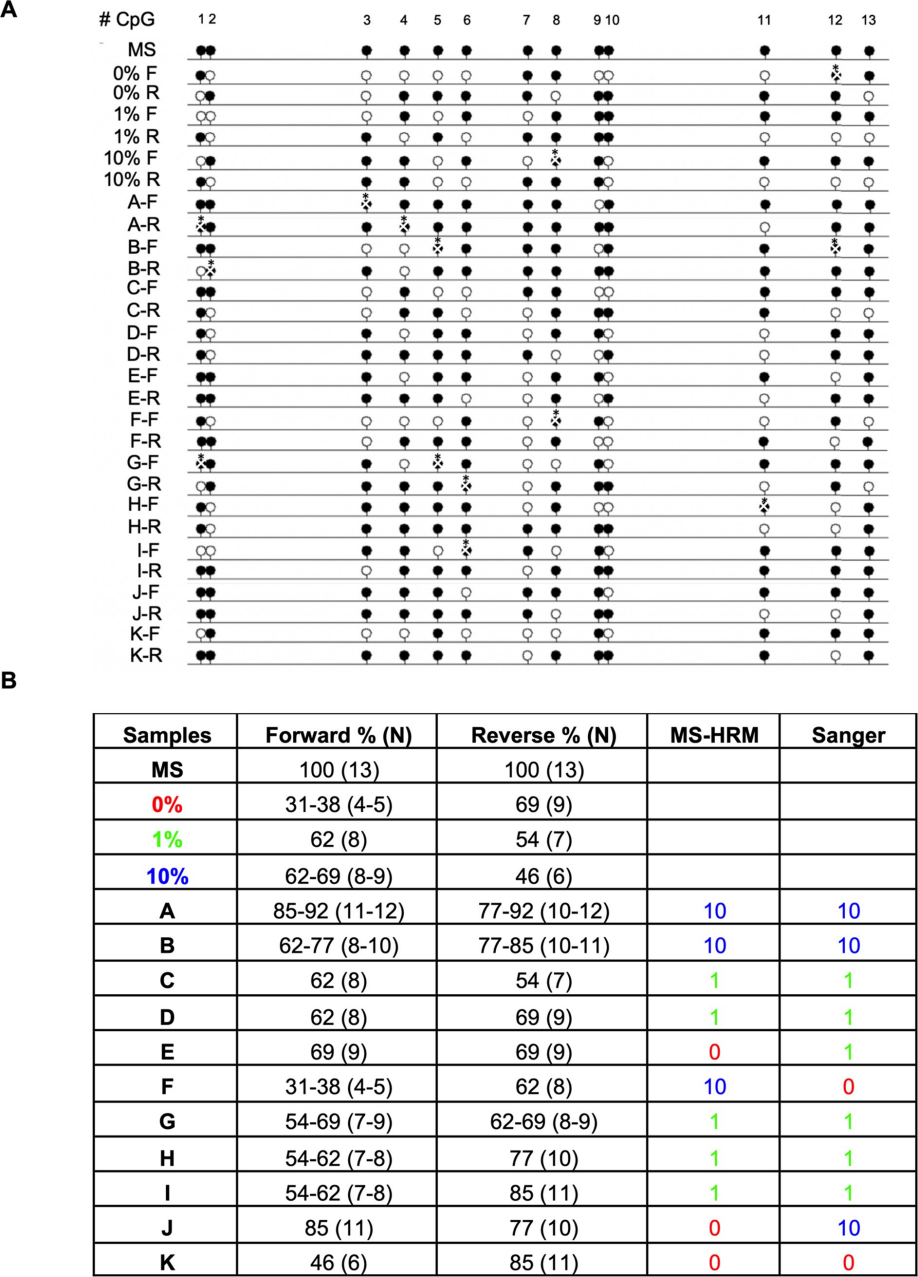
Site-specific LEPR promoter methylation status by Sanger sequencing. (A) Methylation status profiles in WT, MS, human control sets (0, 1 and 10% of DNA methylation) and bisulfite-modified DNA samples by lollipop diagram representation. Methylated CpGs are represented in black lollipops, non-methylated CpGs are in white lollipops, and not defined CpGs are marked with X and asterisk. (B) Percentages of methylation obtained in each allele (forward and reverse sequence) from bisulfite-modified DNA samples and those compared to MS and control set percentages in both MS-HRM and Sanger methods.

Percentages of methylated CpG sites along forward and reverse *LEPR* promoter sequences were calculated to determine the allelic methylation status from MS-HRM products. Sequencing findings exhibited an allelic heterogeneity without similarities between the profiles of DNA methylation in control sets and MS-HRM products (Fig 4A). Percentages of methylated CpGs in both alleles were also different from the same MS-HRM product. Due to this, we divided MS-HRM products in three ranges of methylated CpGs according to the number of methylated CpGs for each allele and as a whole (Methylated CpGs in forward allele of: 0% samples = 31–46, 1% samples = 54–59, and 10% samples = 62–92; and in reverse allele of: 0% samples = 62–85, 1% samples = 54–85, and 10% samples = 77–92), finding that more than 72% of MS-HRM products sequenced had similar methylation status such as DNA samples assayed by MS-HRM technique (Fig 4B).

### Lep-Rb Expression in umbilical vein

**Fig 5:**
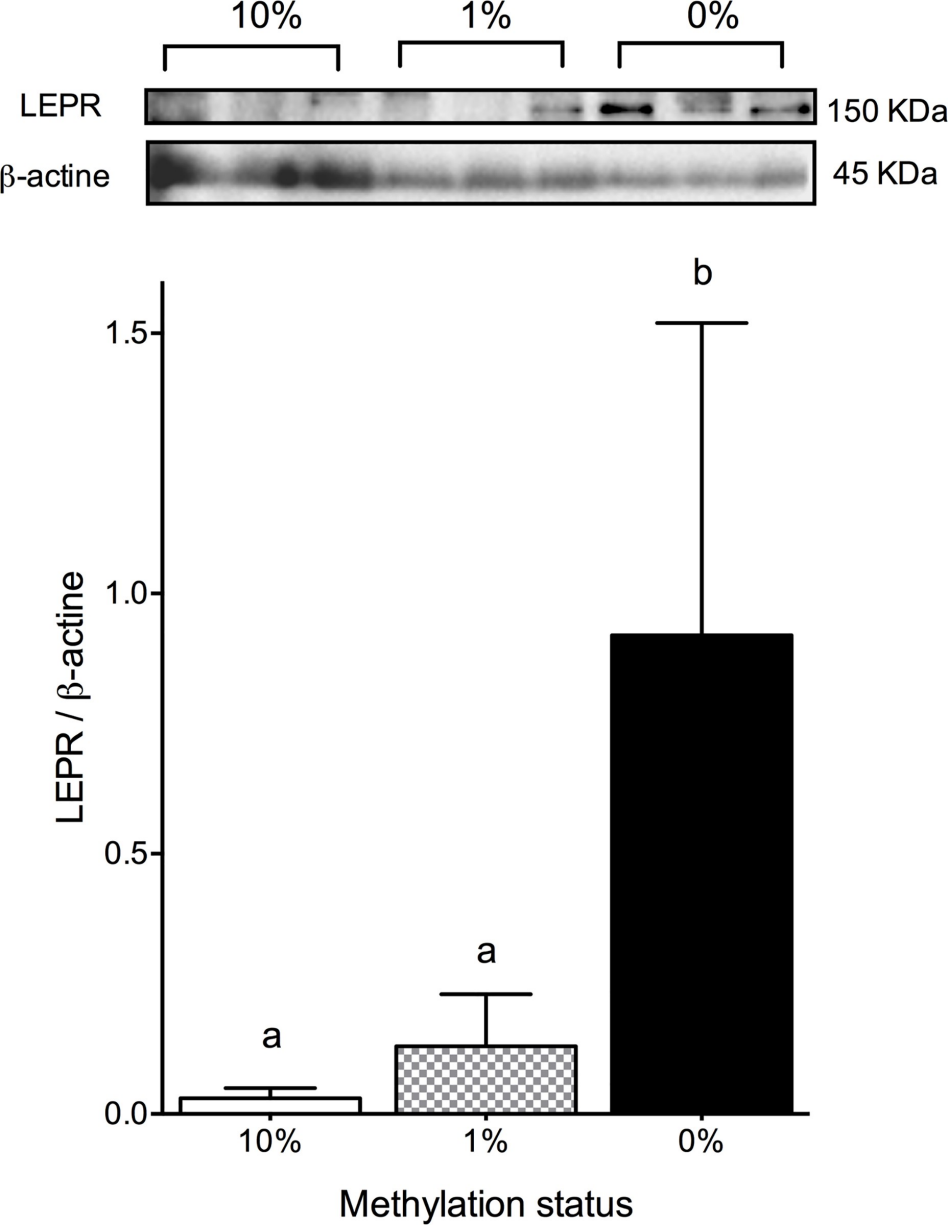
Lep-Rb expression in the umbilical vein from different statuses of gene promoter methylation at term pregnancy. The figure shows a representative Western Blot and the signal quantification of Lep-Rb at three different DNA methylation status (0%, 1%, and 10%). The bars and error bars show the means ± SD of eleven protein extracts samples; difference between means (p < 0.05) was obtained by one-way ANOVA post hoc Tukey’s test and this is shown with different letters over the bars.

To evaluate if protein expression of Lep-Rb was affected by *LEPR* promoter methylation status, we quantified the protein extracts of umbilical vein from the same eleven newborns that were randomly selected to sequence their MS-HRM products. Normalized blots with β-actin expression showed that Lep-Rb decreased according to increased *LEPR* methylation status (Fig 5).

## Discussion

In this study, we examined the association of maternal p-BMI and GWG with DNA methylation of single locus regulatory regions of three obesogenic genes (*GNPDA2*, *PGC1α*, and *LEPR*) in umbilical vein of newborns. *GNPDA2* and *PGC1α* locus showed no DNA methylation variability but, a single locus of *LEPR* promoter showed different status of methylation between samples. Our main finding was that *LEPR* methylation status in this newborn tissue was associated with maternal GWG, showing that a poor weight gain during pregnancy reflects deficient nutritional status associated with increased methylation status of *LEPR* in umbilical vein. Furthermore, low methylation of *LEPR* promoter was related with high expression of Lep-Rb. Additional findings included heterogeneity of *LEPR* methylation status among alleles and umbilical vein samples.

The present study adds to a growing list of clinical and epigenetic evidence supporting the role of perinatal environment in modulation of DNA methylation in newborn tissues [42], suggesting that DNA methylation linked to the pregnant women nutritional status may have specific target genes that may be linked to the phenotype of risk for obesity later in life. SNPs of *GNPDA2*, *PGC1α*, and *LEPR* genes contribute to an increased risk of obesity development in Mexican population [28,30–32], and some evidence strongly suggests that obesity-associated polymorphisms are linked with proximal gene regulation, such as enhancers and promoter regions that are differential methylated [43]. According to our knowledge, this study is the first one examining DNA methylation status in a single locus of *GNPDA2* promoter, and the third one that evaluated the *PGC1α* promoter methylation status in umbilical cord [44,45].

Our results show a differential DNA methylation in a single locus of the *LEPR* promoter associated with GWG, supporting the idea that epigenetic variations linked with intrauterine exposures and DNA methylation among obesogenic genes may have an essential role in the etiology and phenotype of disease [46,47]. Moreover, we found an allelic asymmetry in umbilical cord vein, such as the allelically-skewed DNA methylation in *cis* previously reported by Marzi and *cols*, who attribute it to a tissue-specific genotypic variation [48]. However, our finding adds to the accumulating reports that attribute the allelic asymmetry to an inter-individual variation [25,49], as well as the heterogeneity among cells in the own tissue [50] that may are linked to the nutritional epigenetic programing during fetal development [51].

Maternal malnutrition has previously associated with the variation of DNA methylation in genes of leptin signaling in the offspring [52]. GWG is an indicator of maternal nutritional status and reflects the composed growth of the placenta, uterus, amniotic fluid, maternal blood volume, mammary gland, maternal adipose tissue, and the fetus [53]. Epidemiological data indicates that inadequate GWG is associated to several perinatal complications [54], risk of infant death [55] and later child adiposity [56]. This study provides evidence of a strong correlation between the *LEPR* promoter methylation status in umbilical vein and GWG, regardless the maternal age or unhealthy habits. This result may indicate that this gene is a target of the environmental conditions associated to maternal nutritional status during pregnancy.

The role of leptin receptors in fetal tissues still remains unclear, but six isoforms resulting from alternative splicing of the *LEPR* are known (short isoforms: Lep-Ra, c, d, and f; long isoform: Lep-Rb; soluble isoform: Lep-Re) [57,58]. Lep-Rb is expressed in umbilical cord tissue fully transducing activation signal into cord cells. The short and soluble isoforms are expressed in fetal membranes and umbilical cord blood, being able to regulate the leptin transport in and out of amniotic fluid and blood [59,60]. Our results confirmed a differential Lep-Rb expression in the vein of umbilical cord potentially mediated by the *LEPR* promoter methylation status, supporting the notion that a poor maternal weight gain may promote a differential expression of leptin receptors such as a compensatory mechanism of the fetus [61–64].

Leptin concentration in umbilical cord blood and autocrine placental function through differential leptin receptors expression are being linked with fetal growth, body fat at birth, and childhood growth [65–67]. Indeed in healthy singleton pregnancies, low levels of leptin in umbilical cord blood, high leptin concentration and low expression of both Lep-Ra and Lep-Rb isoforms in placenta have been found in small for gestational age newborns [68]. Moreover, this adipokine is expressed in the fetal side of the placenta greater than in the maternal side, being associated with a higher level of leptin promoter methylation and with a lower expression of the leptin receptor in pregnant women with obesity [69]. This last finding does not contradict our results, conversely, it supports the idea that an excessive or low weight gains during pregnancy may are intrauterine risk factors that through epigenetic mechanisms regulate the leptin system expression, relating them with the long-term programming of energy homeostasis.

Recent evidence is accumulating about *LEPR* regulation in the decidua by specific miRNAs during development and progression of early pregnancy [70]. miRNAs are short molecules of non-coding RNAs which are considered molecules of the epigenetic mechanism that post-transcriptional regulate target genes. Dysregulation of specific miRNAs in placenta and maternal serum have been associated with preeclampsia and maternal obesity, and this may have repercussions on fetal and postnatal growth [71–73]. All this literature makes us think that *LEPR* possibly is a target gene epigenetically programmed during pregnancy [74] that may contribute for the obesogenic phenotype traits, such as differential leptin sensitivity, leading to food intake dysfunction and unregulated reward system functioning, promoting adipogenesis in the future life [75].

Finally, we conducted additional analysis to explore the influence of tobacco smoking, alcohol consumption and illicit drugs use during pregnancy [76] and the *LEPR* promoter methylation status, [77]. However, we found no statistical association, maybe because of the small number of participants who reported those behaviors.

Our study has some limitations, and the biggest one is the relatively small number of participants and the gender lack analysis, which would have added information as a confounding variable for interpreting differential sexual dimorphism of leptin receptors expression [78]. For future, *LEPR*-associated polymorphism studies in umbilical vein have to be made, as the examination of differential DNA methylation in *GNPDA2* and *PGC1α* genes affecting other regulatory regions of the genes must be accomplished. Finally, DNA samples bisulfite-sequencing showed higher than 70% of correlation with the MS-HRM results, having to take the results of allele bisulfite-sequencing for the position of methylation marks cautiously [79].

## Conclusions

In summary, our findings suggest that an inadequate maternal weight gain during pregnancy is associated to *LEPR* promoter methylation in the newborn, affecting receptor expression. Although these disturbances apparently do not have early consequences on newborn size, differential *LEPR* promoter methylation and receptor expression may have repercussions in the individual future responses to food intake, energy signals, and adipogenesis. Determining the prenatal and environmental factors that affect genes and protein expression via DNA methylation are important for the assessment of adverse neonatal outcomes and obesity risk.

## Supporting information

S1 Fig Newborn’s growth indicators and their correlation with maternal nutritional status in the studied subjects. (A-B) BW is positively correlated with maternal p-BMI but not with GWG. (C-D) BL showed no correlation with maternal p-BMI nor with GWG. Statistical difference data (p < 0.01) were obtained by Pearson’s correlation test. p-BMI: pre-pregnancy Body Mass Index; BW: Birth Weight; BL: Birth Length; GWG: Gestational Weight Gain.
(TIFF)

S1 File MS-HRM assays optimization with the primers designed. (PDF)

S2 File Sequence alignments of a single locus of LEPR gene promoter from MS-HRM products. Eleven of MS-HRM products were randomly selected to validate *LEPR* promoter methylation status by Sanger sequencing. (A) Wild-type sequence (WT) of *LEPR* promoter near TSS (Chr1: 65,886,000–65,886,146; USCSC Genome Browser) and its composition of 13 CpGs scattered in 147 nitrogenous bases. The hypothetical methylated sequence (MS) is shown to evidence the methylated CpG positions. Letters, numbers, and signs nomenclatures are at the right size. (B) forward (-F) and reverse (-R) align sequences of MS-HRM products (A to K), WT, MS, and human DNA methylation control sets (0, 1 and 10%).
(PDF)
