# Supplementary figures and images for: Influence of pre-pregnancy body mass index (p-BMI) and gestational weight gain (GWG) on DNA methylation and protein expression of obesogenic genes in umbilical vein
Source: PLoS One. 2019 Dec 3;14(12):e0226010. doi: 10.1371/journal.pone.0226010 (PMC6890247; doi:10.1371/journal.pone.0226010)

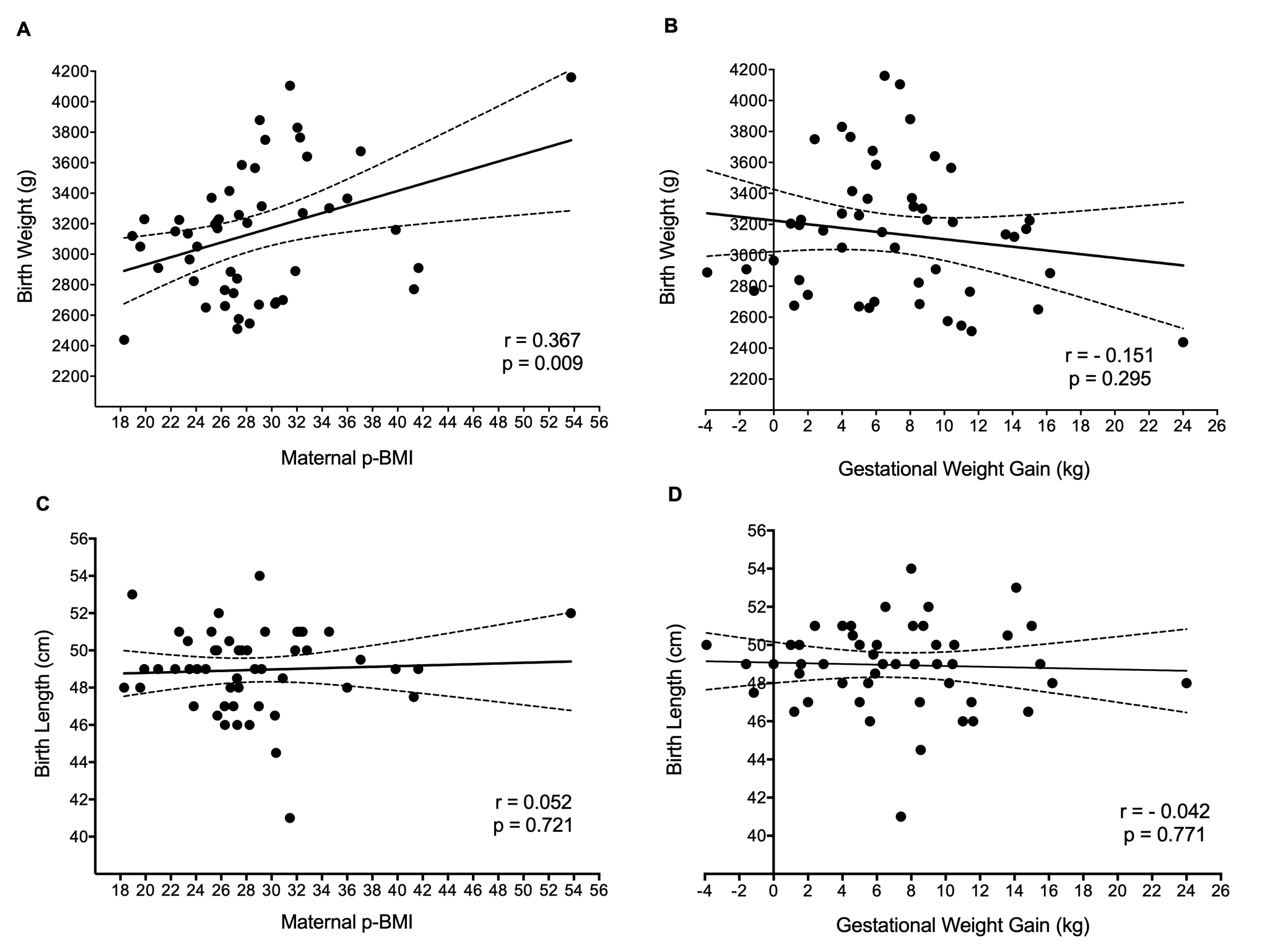

Supplement: S1 Fig — (A-B) BW is positively correlated with maternal p-BMI but not with GWG. (C-D) BL showed no correlation with maternal p-BMI nor with GWG. Statistical difference data (p < 0.01) were obtained by Pearson’s correlation test. p-BMI: pre-pregnancy Body Mass Index; BW: Birth Weight; BL: Birth Length; GWG: Gestational Weight Gain. (TIFF) [file pone.0226010.s001.tiff]
