# Supplementary material for: Influence of pre-pregnancy body mass index (p-BMI) and gestational weight gain (GWG) on DNA methylation and protein expression of obesogenic genes in umbilical vein
Source: PLoS One. 2019 Dec 3;14(12):e0226010. doi: 10.1371/journal.pone.0226010 (PMC6890247; doi:10.1371/journal.pone.0226010)
Supplement: S1 File — (PDF) [file pone.0226010.s002.pdf]

A) ***GNPDA2* primers**

Forward: 5’-ATTCGGATGTAGATAAAGGCGTAG-3’

Reverse: 5’-CTCCTCGCGTCTCACCTCAA-3’

B) Figures representing the different annealing temperatures used for MS-HRM assay optimization with *GNPDA2* primers.

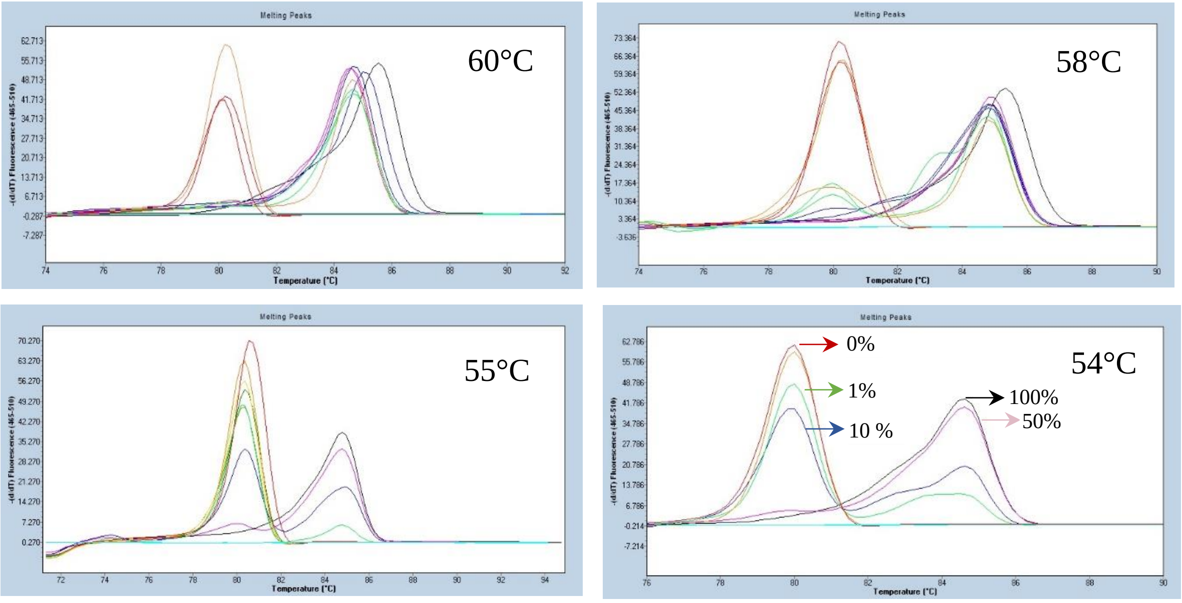

C) Melting temperatures obtained with the annealing temperature of 54°C.

**54°C**	**Assay 1**		**Assay 2**		**Assay 3**		
**Control set**	**1**	**2**	**1**	**2**	**1**	**2**	**CV (%)**
100%	86.7	86.4	85.4	85	85.8	85.4	0.76
50%	86.3	86.3	84.9	84.9	85.2	85.2	0.77
10%	86.3	86	84.8	84.8	85	85.1	0.76
1%	81.6	81.6	80.4	80.3	80.7	80.7	0.71
0%	81.6	81.6	80.4	80.3	80.7	80.7	0.71

CV: coefficient of variation

A) ***LEPR* primers**

Forward: 5’-TTCGCGAGTTAGGGGAGGAG-3’

Reverse: 5’-ACCCGACCTCGCTACTCAAA-3’

B) Figures representing the different annealing temperatures used for MS-HRM assay optimization with *LEPR* primers.

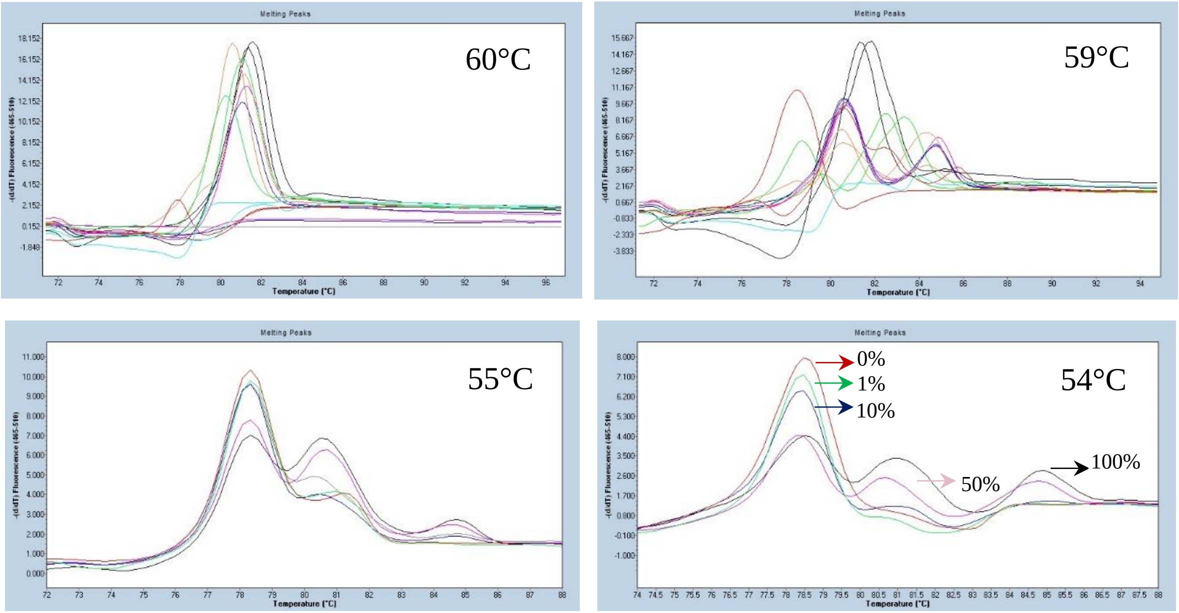

C) Melting temperatures obtained with the annealing temperature of 54°C.

**54°C**	**Assay 1**		**Assay 2**		**Assay 3**		
**Control set**	**1**	**2**	**1**	**2**	**1**	**2**	**CV (%)**
100%	84.6	84.6	85.4	84.7	85.2	85.6	0.51
50%	80.8	80.6	80.4	80.7	80.7	80.7	0.17
10%	80.4	81.4	81.3	81.3	80.6	80.5	0.57
1%	78	78	78.1	77.8	78.4	78.4	0.31
0%	77.7	77.7	77.7	77.8	78.2	78	0.26

CV: coefficient of variation

A) *PGC1α* primers

Forward: 5’-TTCGCGAGTTAGGGGAGGAG-3’

Reverse: 5’-ACCCGACCTCGCTACTCAAA-3’

B) Figures representing the different annealing temperatures used for MS-HRM assay optimization with *PGC1α* primers.

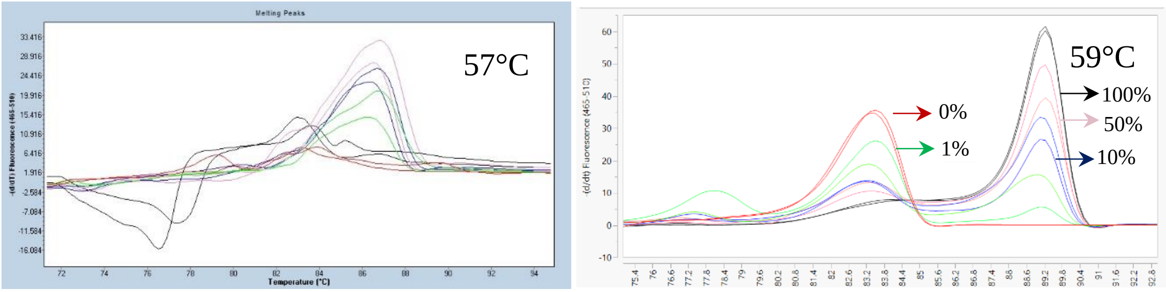

C) Melting temperatures obtained with the annealing temperature of 59°C.

**59°C**	**Assay 1**		**Assay 2**		**Assay 3**		
**Control set**	**1**	**2**	**1**	**2**	**1**	**2**	**CV (%)**
100%	89.2	89.2	89.1	89.2	89.3	89.2	0.07
50%	89.2	89.3	89.2	89.1	89.1	89.2	0.08
10%	89.1	89.1	88.9	89	89.1	89	0.09
1%	83.6	83.5	83.6	83.7	83.8	83.7	0.13
0%	83.5	83.4	83.6	83.7	83.6	83.6	0.12

CV: coefficient of variation
