# Supplementary material for: Influence of pre-pregnancy body mass index (p-BMI) and gestational weight gain (GWG) on DNA methylation and protein expression of obesogenic genes in umbilical vein
Source: PLoS One. 2019 Dec 3;14(12):e0226010. doi: 10.1371/journal.pone.0226010 (PMC6890247; doi:10.1371/journal.pone.0226010)

A

```
# CpG:       1  2                                                        3               4              5            6                          7           8                 9 10
WT: 5´- TCCGCGAGCTAGGGGAGGAGCTCTGCATTGTCTGGGGCGGGGCTTCGAGTAGCGGTAGCGAGGTCTGGAGCCGCAGGCGCTGCCTCCGCG
        |:++++||:|||||||||||:|:||:|||||:|||||++|||:||++|||||++||||++||||:|||||:++:|||++:||::|:++++
MS: 5´- TTCGCGAGTTAGGGGAGGAGTTTTGTATTGTTTGGGGCGGGGTTTCGAGTAGCGGTAGCGAGGTTTGGAGTCGTAGGCGTTGTTTTCGCG

                                                 11                     12         13
        AGGTAGGGGAGGAGCTCTGTACTGTCAGGGGCGGGGCTCTGAGTAGCGAGGCCGGGT -3´
        ||||||||||||||:|:||||:|||:|||||++|||:|:|||||||++|||:++|||
        AGGTAGGGGAGGAGTTTTGTATTGTTAGGGGCGGGGTTTTGAGTAGCGAGGTCGGGT -3´
```

WT: Wild type
MS: Methylated sequence
: C converted to T
+ CpG site
| Preserved site
Oligonucleotides
Underlined: SP1 transcription factor binding site

B

Adenine   Thymine   Cytosine   Guanine

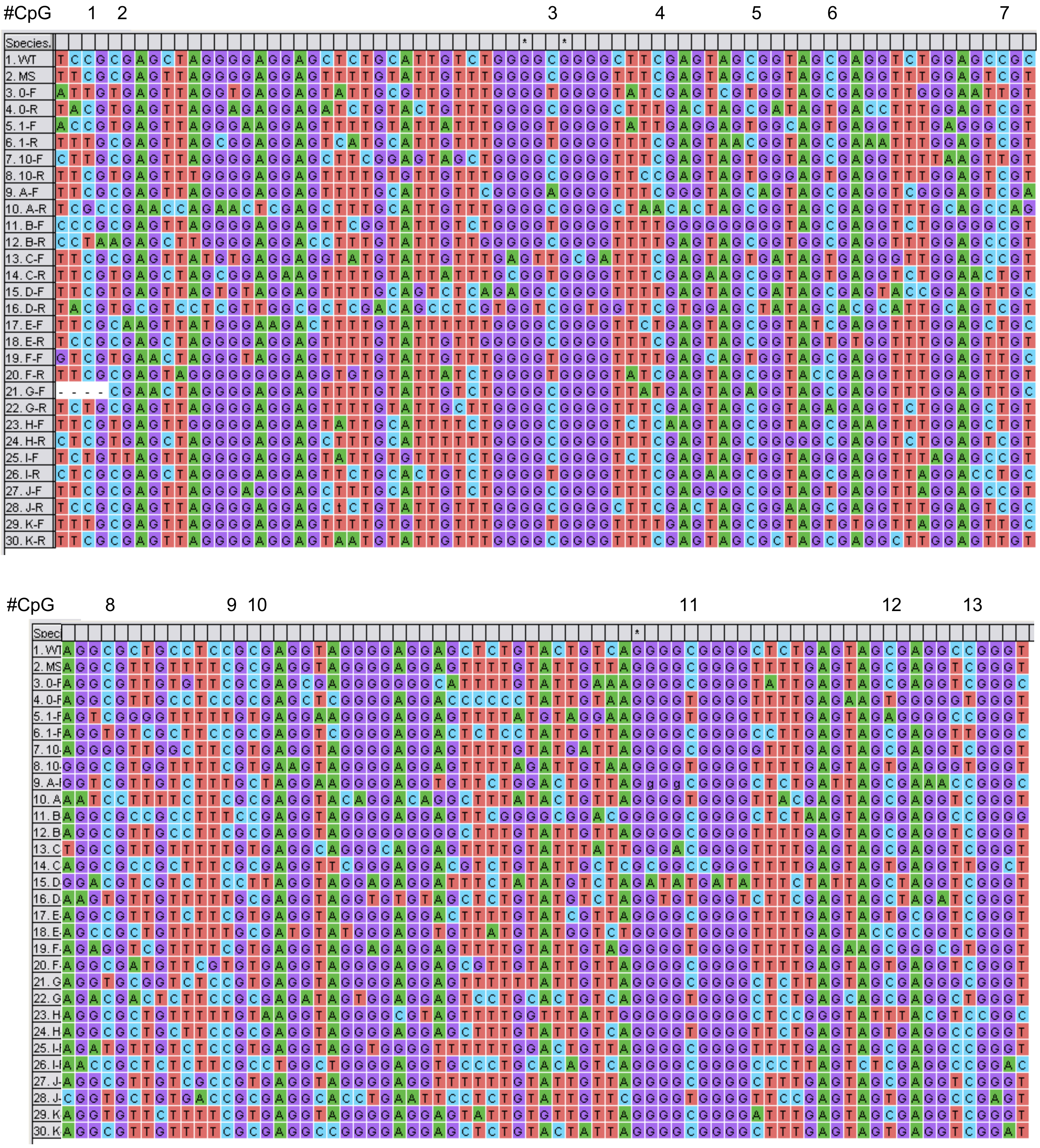

Supplement: S2 File — Eleven of MS-HRM products were randomly selected to validate LEPR promoter methylation status by Sanger sequencing. (A) Wild-type sequence (WT) of LEPR promoter near TSS (Chr1: 65,886,000–65,886,146; USCSC Genome Browser) and its composition of 13 CpGs scattered in 147 nitrogenous bases. The hypothetical methylated sequence (MS) is shown to evidence the methylated CpG positions. Letters, numbers, and signs nomenclatures are at the right size. (B) forward (-F) and reverse (-R) align sequences of MS-HRM products (A to K), WT, MS, and human DNA methylation control sets (0, 1 and 10%). (PDF) [file pone.0226010.s003.pdf]
